# Supplementary figures and images for: Using camera-guided electrode microdrive navigation for precise 3D targeting of macaque brain sites
Source: PLoS One. 2024 May 28;19(5):e0301849. doi: 10.1371/journal.pone.0301849 (PMC11132476; doi:10.1371/journal.pone.0301849)

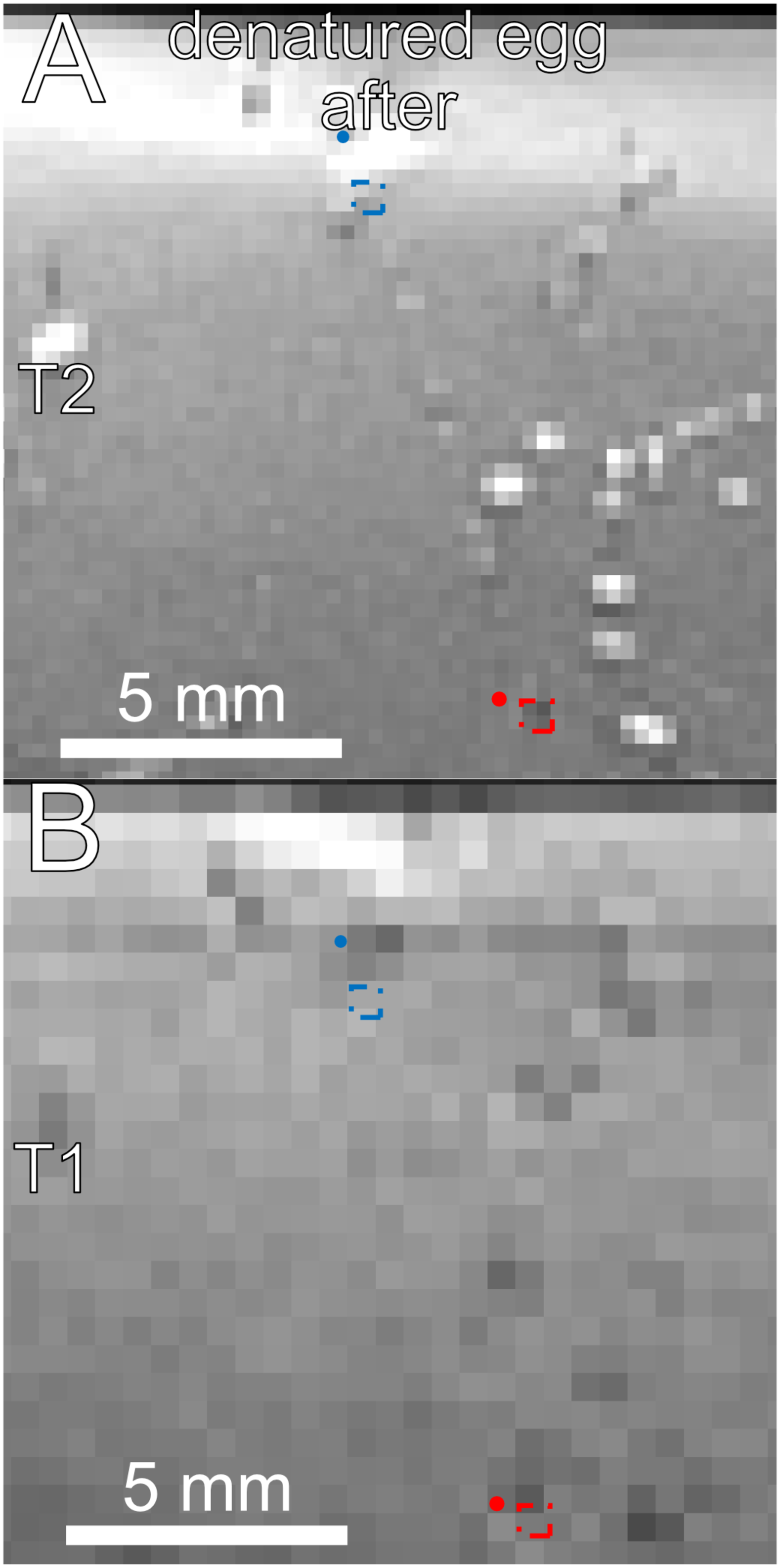

Supplement: S1 Fig — Planned (dots) and evaluated (squares) locations of the guide tube penetration entry point and iron deposits are added in blue and red, respectively. Due to the higher resolution and signal quality, T2-imaging was used for precise localization of iron deposits and guide tube penetration marks. In A, the electrode tract connecting the two squares can be identified. A is a T2-weighted image; B is a T1-weighted image. A & B were taken after the iron deposition. (TIF) [file pone.0301849.s001.tif]

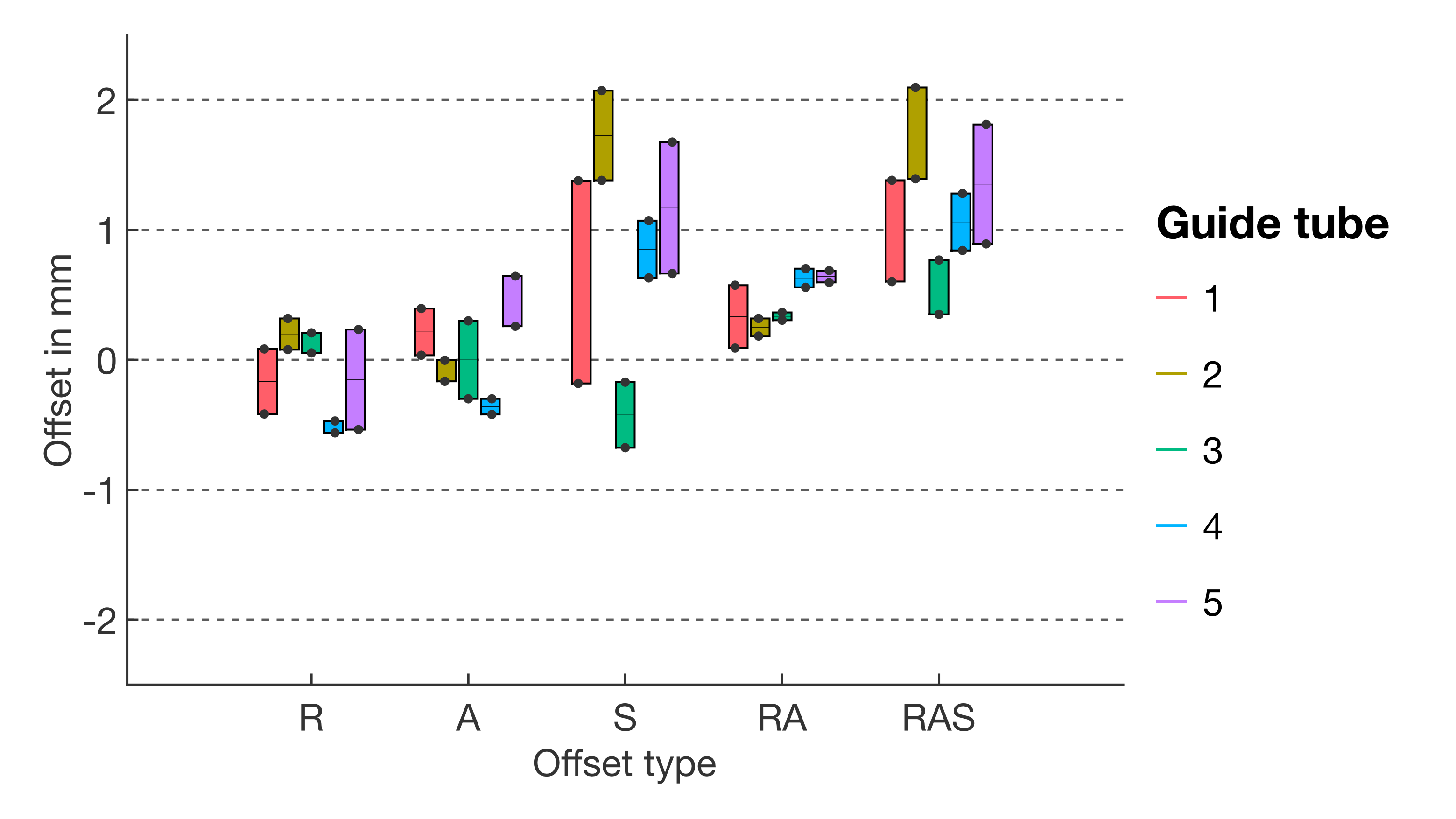

Supplement: S2 Fig — R is the offset to the right, A to the anterior and RA the 2-dimensional distance in the RA-plane. S is the offset in the superior direction and RAS depicts the 3-dimensional distance. (TIF) [file pone.0301849.s002.tif]

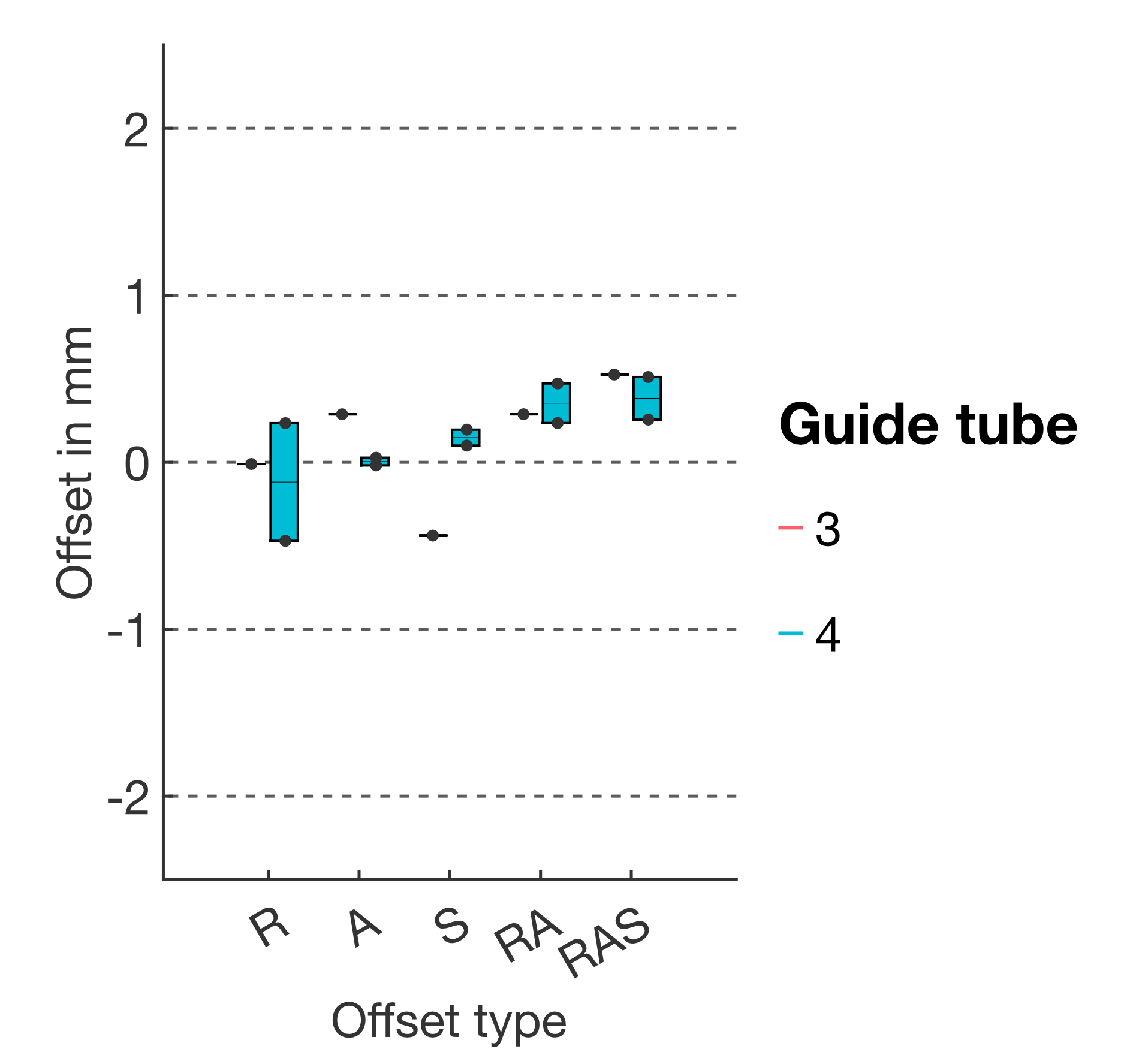

Supplement: S3 Fig — R is the offset to the right, A to the anterior and RA the 2-dimensional distance in the RA-plane. S is the offset in the superior direction and RAS depicts the 3-dimensional distance. (TIF) [file pone.0301849.s003.tif]

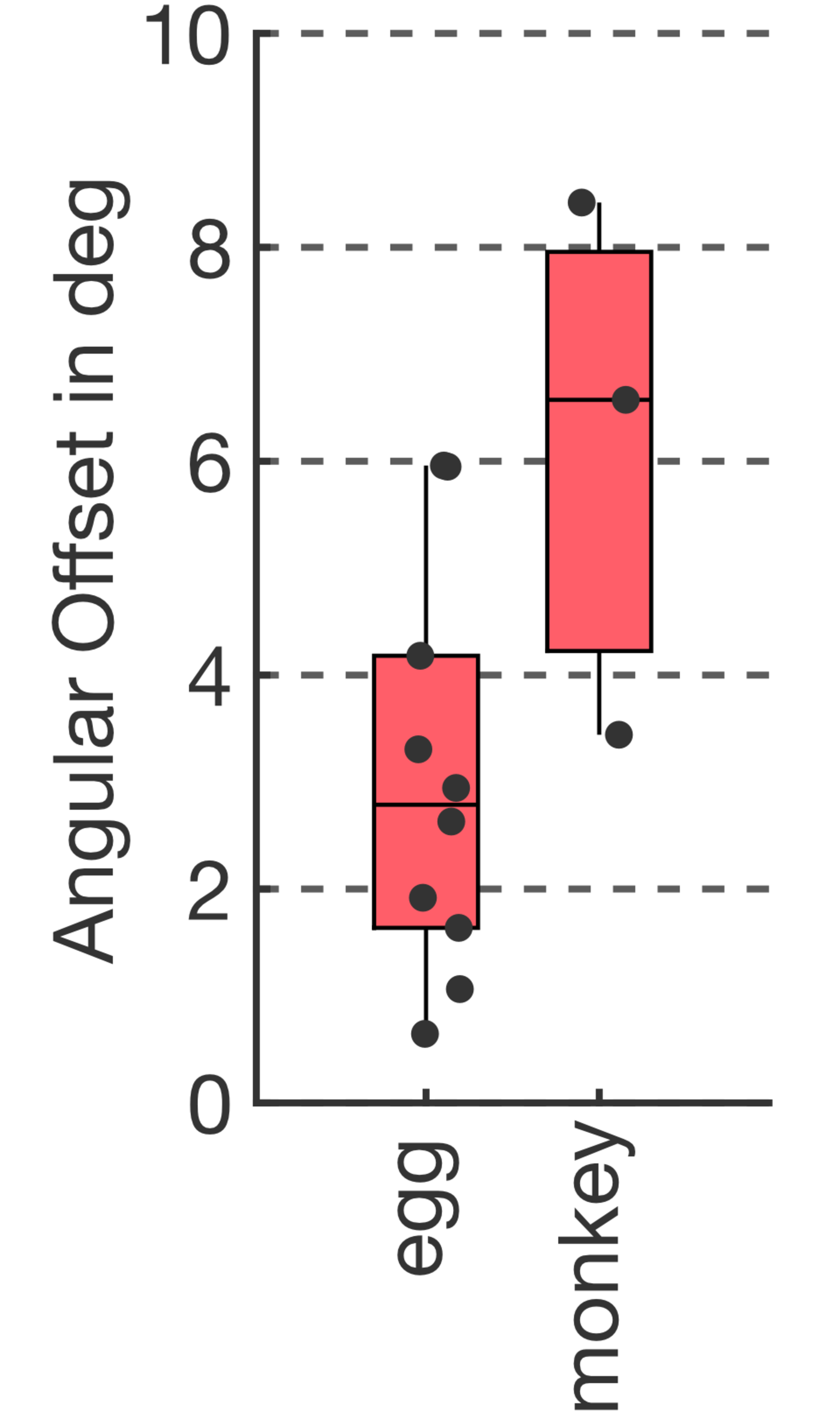

Supplement: S4 Fig — (TIF) [file pone.0301849.s004.tif]

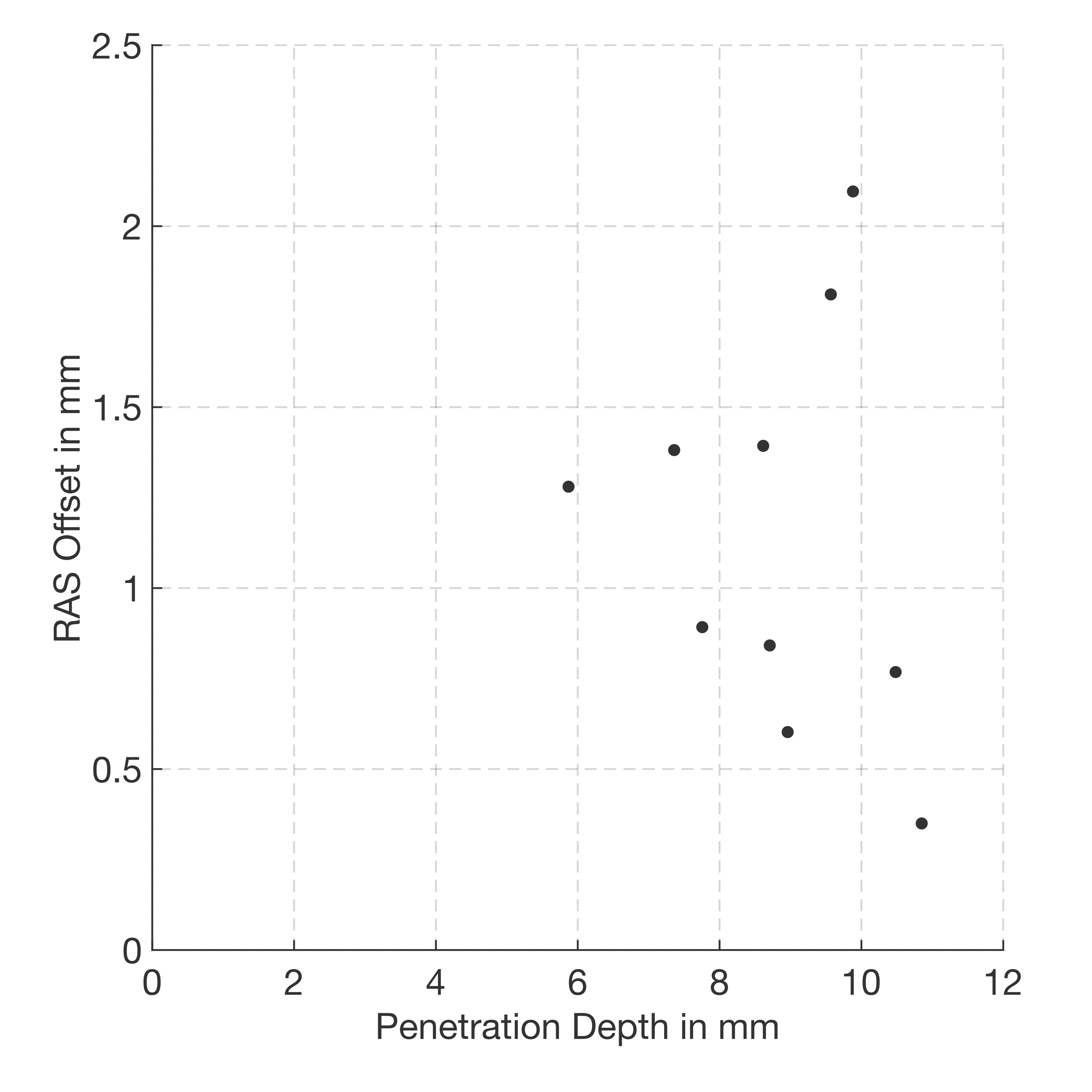

Supplement: S5 Fig — (TIF) [file pone.0301849.s005.tif]
